# Degree of satisfaction of healthcare workers (HCW) with the training received on the use of personal protective equipment (PPE) for the care of patients with suspected Ebola virus disease (EVE): final results

**DOI:** 10.1186/2047-2994-4-S1-P7

**Published:** 2015-06-16

**Authors:** I Tenza Iglesias, JG Mora Muriel, EJ Silva Contreras, JL Mendoza García, CO Villanueva Ruiz, J Barrenengoa Sañudo, E López Gonzalez, J Bravo Miro, J Sánchez Payá

**Affiliations:** 1 Hospital General Universitario de Alicante, Alicante, Spain

## Introduction

The probability to treat patients with suspected EVE has determined that the training of HCW in the use of PPE is encouraged.

## Objectives

To quantify the degree of satisfaction, and its associated factors, of HCW who have attended the workshop on the use of PPE.

## Methods

Observational cross-sectional study. The number of professionals who have attended the workshops is 582 (groups of 8-10 people). The explanatory variables are: implementation period (consolidated / initial), day of the week when workshop was attended (Monday-Tuesday-Wednesday / Thursday-Friday), time frame (13-15 h / 15-17 h), usual place of work (emergency / other), age (=45 years old), sex (male / female), stratum of the participant (physicians or others) and stratum of the speaker (head staff physician / resident physician). To quantify satisfaction, scale from 0 to 10 has been used, and the variable "High Degree of Satisfaction" (HDS) has been defined when satisfaction was scored 8, 9 or 10. To study the association between explanatory variables and HDS we used the chi-square test, and to quantify the magnitude of the association we calculated adjusted odds ratio (ORa) and its 95% confidence intervals (95% CI) with a multiple logistic regression model.

## Results

86.8% (505/582) of the professionals show a HDS. The variables independently associated with a HDS were: period (consolidated, 88.5%, vs initial, 75.6%; ORa = 3.0 (1.3-6.8); stratum of the participant (physicians, 89.8%, vs others, 84.7%; ORa = 2.6 (1.2-5.5); stratum of the speaker (staff physician, 82.9%, vs resident physician, 98.3%, ORa = 0.4 (0.2-0.8).

## Conclusion

The degree of satisfaction of HCW who attended workshops is very high, and it is associated with the variables period of realization, workplace and stratum of the speaker. Knowing the satisfaction of professionals attending training programs is an strategic element in the planning of healthcare-associated infections prevention programs.

## Disclosure of interest

None declared.

